# Health policy and systems research collaboration pathways: lessons from a network science analysis

**DOI:** 10.1186/s12961-017-0241-5

**Published:** 2017-08-28

**Authors:** Krista M. English, Babak Pourbohloul

**Affiliations:** 1 Complexity Science Lab, School of Population & Public Health, University of British Columbia, Vancouver, British Columbia Canada; 2 Institute of Resources, Environment and Sustainability, University of British Columbia, Vancouver, British Columbia Canada

**Keywords:** Health policy, Systems research, Low- and middle-income countries, Co-authorship networks, Capacity-building

## Abstract

**Background:**

The 2004 Mexico Declaration, and subsequent World Health Assembly resolutions, proposed a concerted support for the global development of health policy and systems research (HPSR). This included coordination across partners and advocates for the field of HPSR to monitor the development of the field, while promoting decision-making power and implementing responsibilities in low- and middle-income countries (LMICs).

**Methods:**

We used a network science approach to examine the structural properties of the HPSR co-authorship network across country economic groups in the PubMed citation database from 1990 to 2015. This analysis summarises the evolution of the publication, co-authorship and citation networks within HPSR.

**Results:**

This method allows identification of several features otherwise not apparent. The co-authorship network has evolved steadily from 1990 to 2015 in terms of number of publications, but more importantly, in terms of co-authorship network connectedness. Our analysis suggests that, despite growth in the contribution from low-income countries to HPSR literature, co-authorship remains highly localised. Lower middle-income countries have made progress toward global connectivity through diversified collaboration with various institutions and regions. Global connectivity of the upper middle-income countries (UpperMICs) are almost on par with high-income countries (HICs), indicating the transition of this group of countries toward becoming major contributors to the field.

**Conclusions:**

Network analysis allows examination of the connectedness among the HSPR community. Initially (early 1990s), research groups operated almost exclusively independently and, despite the topic being specifically on health policy in LMICs, HICs provided lead authorship. Since the early 1990s, the network has evolved significantly. In the full set analysis (1990–2015), for the first time in HPSR history, more than half of the authors are connected and lead authorship from UpperMICs is on par with that of HICs. This demonstrates the shift in participation and influence toward regions which HPSR primarily serves. Understanding these interactions can highlight the current strengths and future opportunities for identifying new strategies to enhance collaboration and support capacity-building efforts for HPSR.

## Background

The Mexico Ministerial Statement for the Promotion of Health (the Mexico Declaration) [1], and subsequent World Health Assembly resolutions, proposed a concerted global programme of work to support the development of health policy and systems research (HPSR). This included coordination across partners and advocates for the field of HPSR to monitor the development of the field, while promoting decision-making power and implementing responsibilities in low- and middle-income countries (LMICs) [1–3].

Bibliometric analysis of HPSR provides a systematic and scientific means of monitoring this development. This task has been carried out by a number of groups in recent years [4, 5], including the authors of this paper [6]. These results have demonstrated that great strides have been made to support and ensure meaningful inclusion of LMICs in HPSR. While lead authorship from LMICs is increasing and outpacing the growth in lead authorship in life and biomedical sciences (PubMed) in general, LMIC authors are significantly under-represented in terms of absolute number of HPSR publications on topics relevant to, and including, LMICs.

Building on this understanding, questions remain regarding the intricate collaborative interactions that shape these trends. Understanding these interactions can highlight the current strengths and future opportunities for identifying new strategies to enhance collaboration and support increased LMIC contribution to HPSR.

To address this, a special framework is required. This framework must simultaneously capture the contributions of individuals (e.g. authors, policymakers, implementers, institutions) in the HPSR literature (micro-level factors), as well as the national, regional or global level trends (macro-level factors). Recent advances in network science have contributed to the development of a framework that allows us to analyse these micro- and macro-level trends as well as other dynamic complexities.

The digitisation of publications and the databases that house them have propelled bibliometric studies to attempt to capture network structures from authors’ names, affiliations and geospatial distribution. In recent years, massive databases, at various levels of granularity, have become readily available for analysis. New methods for analysis have provided inspiration for identifying new metrics and furthering our understanding of the significance and relative contribution of authors, institutions, as well as regional and/or multidisciplinary collaborations. The core concept behind this network analysis approach is based on developments in the physics and computer science communities over the past decade [7–9].

## Methods

**Fig. 1:**
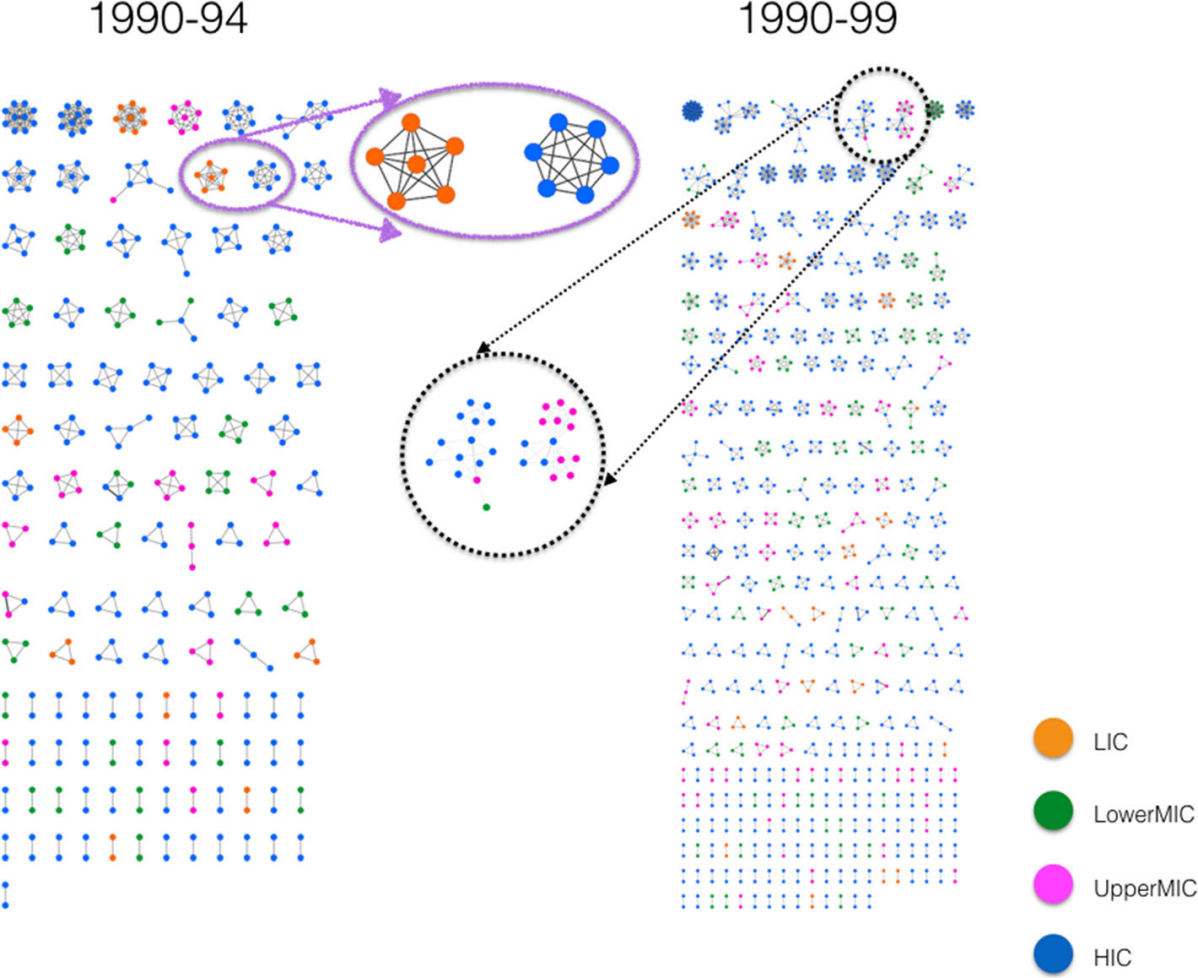
Health policy and systems research co-authorship networks from 1990 to 1994 (left panel) and 1990 to 1999 (right panel). Node colour represents the economic classification of first-authors’ country, as per the World Bank. The left inset shows the small disjoint chains (SDC) prior to 1994, where each chain is comprised of authors from the same economic region, and very often, from the same institution. The right inset shows the authors becoming gradually more connected, yet still considered to have SDC structure. There were 378 nodes in 1990–1994 and 1119 nodes in 1990–1999. *Orange colour* indicates low-income countries (LICs), *green* for lower middle-income countries (LowerMICs), *pink* for upper middle-income countries (UpperMICs) and *blue* for high-income countries (HICs)

We explore a network representation of co-authorship data, hereafter referred to as a co-authorship network. This network is comprised of nodes and edges; each node represents an author who has co-authored at least one HPSR publication, while each edge (link) is represented by a line connecting two nodes and corresponds to publication(s) that were co-authored by those two authors (nodes) (right inset, Fig. 1). The co-authorship networks provide compelling insights into the current state of collaboration within the discipline, between regions and over time.

A co-authorship network can help identify efficient opportunities to strengthen the research capacity in LMICs through international collaborations. The networks can also demonstrate both the gaps and emerging topics within health policy and systems research, facilitating oversight for regional planning to ‘stay ahead of the curve’ by building home-grown capacity relevant to tomorrow’s needs. Similarly, researchers, may identify strategies to maximise their scientific contribution and/or influence on policy decision-making.

A co-authorship network captures collaboration patterns between authors. The type, frequency, distance and number of collaborations determine the pace at which the discipline advances. Co-authors are identified from bibliometric data that have been narrowed to the specific field of study. Additional information contained within the database may enrich the networks and reveal other interesting features about the collaborations. Identifying these patterns over time facilitates our understanding of the dynamic interactions and provides an opportunity to identify strengths and challenges in the HPSR co-authorship network.

PubMed was used to study the network of contributors to the HPSR literature. PubMed is a vast resource of literature relevant to the life and biomedical sciences, including more than 26 million citations, as of August 2016. It has twice as many health policy-relevant publications as the next largest collection [6].

Details of our data collection and processing approach was reported in a previous publication (please see [6]). In summary, we used a high-level keyword search strategy to identify the literature relevant to HPSR and ensure inclusivity. Additional terms and keywords can be added to refine the search or learn more about sub-groups under the HPSR umbrella. The syntax of the high-level keyword search strategy used the logical Boolean operators “AND” and “OR”: (health AND policy) OR “health system*”. While the specific topic of the paper may be related to any area within the scope of HPSR, this strategy assumes that papers related to HPSR would have the words ‘health’ and ‘policy’ or ‘health system(s)’ somewhere in the text. PubMed includes a prescribed set of filters to identify specific topics related to clinical queries and medical genetics [10]. The exclusion criteria can be applied to the search strategy using the Boolean operator, “NOT”, thereby removing the irrelevant clinical literature [11]. The *species* filter was applied to restrict the results to human studies [12], resulting in approximately 85,000 HPSR publications.

The HPSR literature was further refined to a cohort of publications that captured topics relevant to LMICs, resulting in a subset of approximately 7000 from the above 85,000 HPSR publications. This subset serves as the basis for much of the analysis that follows.

To identify the collection of papers with its main topic focused on an issue relevant to a LMICs, we first performed the keyword search strategy to identify the subset of publications relevant to health policy and systems research. We then used the title and abstract sections, denoted by the tag “Title/Abstract [TIAB]”, as it is intended to most concisely describe the main focus and purpose of a paper. Therefore, HPSR publications with a main focus relevant to LMICs can be efficiently identified by limiting the search to the list of 135 LMICs and synonyms for “developing country” that appear in the title and abstract [13]. Keywords (topics) may also be included here, but without mention of an LMIC, it would be difficult to determine whether the topic is specifically relevant to LMICs or of a more general HPSR issue relevant to high-income countries (HICs).

The networks were produced by Cytoscape, an open-source software platform for visualising complex networks [14]. The input to this software comprised of compiled files downloaded from PubMed as described above. The visualisation techniques used to show the networks can include millions of nodes and edges. This scalability is advantageous when studying networks that are increasing in size over time, such as the emerging and expanding discipline of HPSR.

Interpretation of a co-authorship network structure requires careful consideration, illustrated through the inset in Fig. 1. Let us assume that six individuals co-author a paper. In this case, these individuals are represented by six nodes in the network, and since they are all co-authors on the same paper, each pair of them must be connected to one another with an edge, resulting in 6 × (6–1)/2 = 15 edges between them (see left inset in Fig. 1). Similarly, if a paper is co-authored by 10 authors, then the 10 nodes representing these authors must be connected to one another by 10 × (10–1)/2 = 45 edges. Therefore, while each author is uniquely represented by a node in the network, a paper may be represented by multiple edges depending on the number of co-authors on that paper.

On the other hand, let us assume that two authors co-authored just one paper. In this case, the two authors are represented by two nodes, while the edge between them represents the sole co-authored publication. Similarly, let us assume that two authors co-authored 15 papers together. In this case, again, the two authors are represented by two nodes; however, they are connected by a thicker edge representing all 15 publications co-authored by them. As such, the thickness of an edge depends on the number of papers co-authored between two authors (nodes) within a given time interval; the higher the number of co-authored papers, the thicker the edge connecting these two nodes.

## Results and Discussion

### Contribution of different economic regions to the HPSR literature

Prior to 2014, PubMed only required the first author of a paper to provide their institutional affiliation as part of author bibliographic data. The first author’s affiliation was used as a proxy to represent author’s country of residence. Given that only one institution/country is assigned to each publication in PubMed, this affiliation was attributed to the same paper, regardless of the subsequent authors’ affiliations. While this facilitates capturing the global connectivity of co-authors, it limits our ability to analyse all co-authors’ countries. Despite this limitation on the secondary analysis of the database, important observations can be summarised with regards to regional contribution to the HPSR literature, as it depends, by and large, on the first-authors’ affiliations.

In addition to the global behaviour of the HPSR co-authorship network, the contribution of different economic regions may be examined. The World Bank’s 2016 fiscal year country economic classification was applied retrospectively to all previous years. This classification includes low-income countries (LICs; with a gross national income (GNI) of US$1025 or less in 2015), lower middle-income countries (LowerMICs; with a GNI between US$1026 and US$4035), upper middle-income countries (UpperMICs; with a GNI between US$4036 and US$12,475), and HICs (with a GNI greater than US$12,476) [15]. The specific colour codes used in the following figures correspond to different World Bank economic regions.

To analyse the HPSR publications, systematically, we divided the period from 1990 to 2015 into five consecutive 5-year intervals; the last interval covers 6 years to include 2015, the last year before conducting this study.

Figure 1 (left panel) shows the HPSR co-authorship network for the first time interval between 1990 and 1994, which represents 378 authors (nodes). The network is comprised of small groups of authors, ranging from 2 to 10, and who collaborate in clusters that are separate from one another, referred to as small disjoint chains or small disjoint components (SDCs). Given the very low number of co-publications between authors during this interval, almost every SDC in this figure is limited to one economic region, i.e. all nodes within each SDC have the same colour. This corresponds to the early stage of the formation of the HPSR literature, when many groups and individuals work in isolation. This time interval also experienced a low number of publications (five or fewer) per person.

Collaboration and co-authorship between individuals is not an isolated activity; it spans across their professional careers. As such, it is important to view and analyse their collective behaviour, in a cumulative manner, over time. To achieve this objective, we present the *cumulative networks* for the subsequent intervals after 1994. In other words, we investigate the network behaviour for the intervals of 1990–1999, 1990–2004, 1990–2009 and finally, 1990–2015, by incrementally adding new nodes and edges to the existing network from previous interval(s).

Figure 1 (right panel) shows the network for the interval 1990–1999, with 1119 authors contributing to the HPSR literature. An increase in the number of publications and participation of more authors during this extended interval marks the beginning of formation of clusters that are composed of authors from different regions (see right inset in Fig. 1). Despite this evolution, the global structure of the network remained, by and large, disconnected and only comprised of SDCs. In addition, while only papers that focus on a topic relevant to LMICs have been included, the majority of first authors are from HICs, while very few are from LICs. Furthermore, during the initial stage of HPSR development, HIC nodes play a prominent role in binding the network together.

**Fig. 2:**
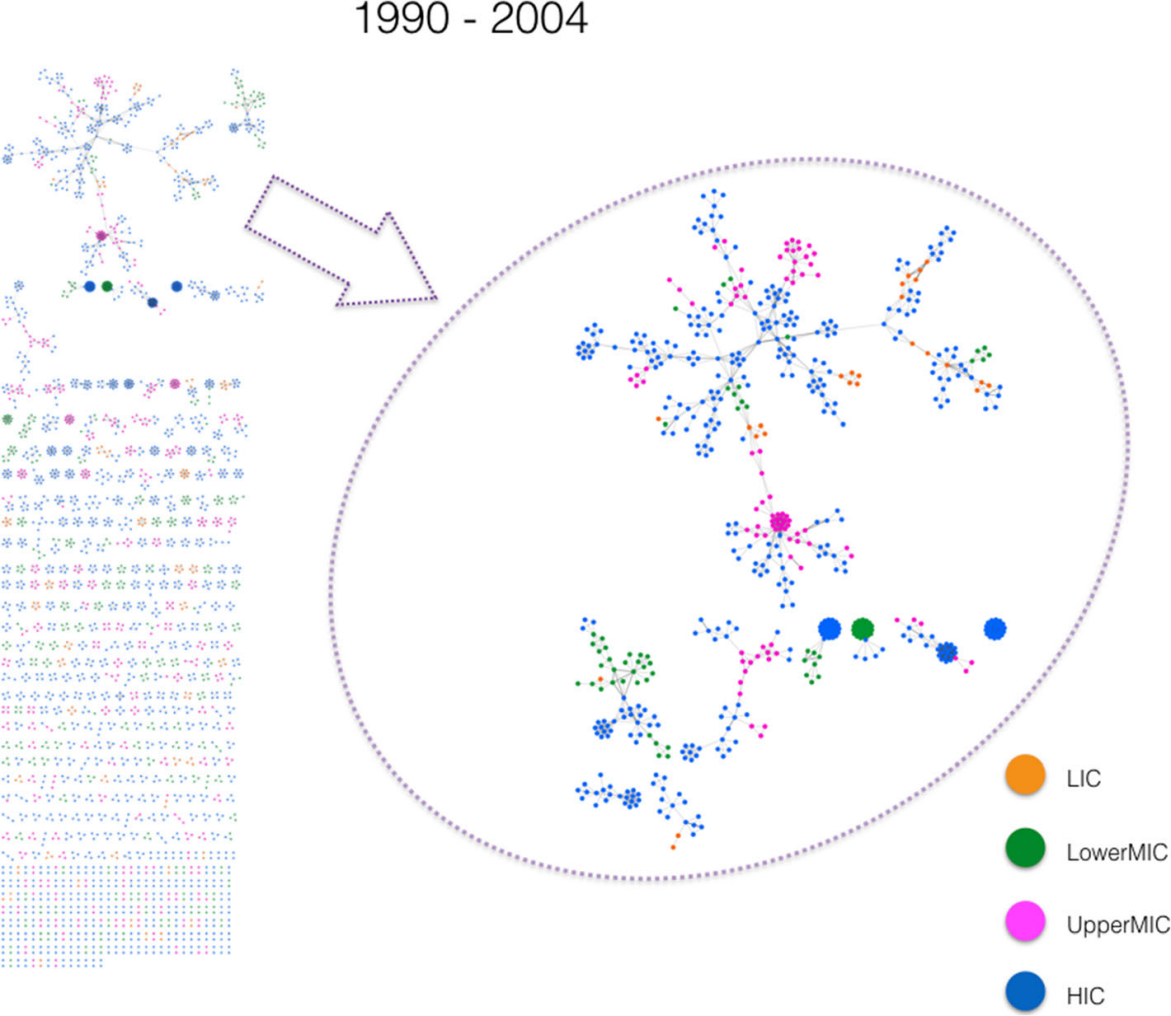
Health policy and systems research co-authorship network structure from 1990 to 2004. In contrast to previous intervals, a large connected component (LCC) is formed during this interval (upper part of the left panel). The right panel shows an enlarged view of this LCC, which shows various sub-structures, may suggest the beginning of a broad and heterogeneous pattern of collaboration among co-authors. Colour codes are the same as Fig. 1. Of a total of 2887 nodes in this interval, 2281 contribute to the formation of SDCs and 606 belong to the LCC. *Orange colour* indicates low-income countries (LICs), *green* for lower middle-income countries (LowerMICs), *pink* for upper middle-income countries (UpperMICs) and *blue* for high-income countries (HICs)

The cumulative interval between 1990 and 2004 marks an important transition in overall (global) connectivity of the HPSR co-authorship network. For the first time, the volume and diversity of collaboration grew to 2887 authors. This network size allowed for the formation of the largest connected component (LCC). This component is magnified within a dashed ellipse in Fig. 2. The formation of LCCs is indicative of the ability of co-authors to work collaboratively beyond their previously-isolated SDC and establish new ties with authors in other SDCs over time. A closer look at the LCC reveals that, at this initial phase, the dendritic structure of LCC remains fragile and the connectivity of the component depends on a few critical edges (co-authored papers). While 606 (21%) nodes belong to the LCC in this interval, the majority of nodes (2281 or 79%) are still SDCs.

**Fig. 3:**
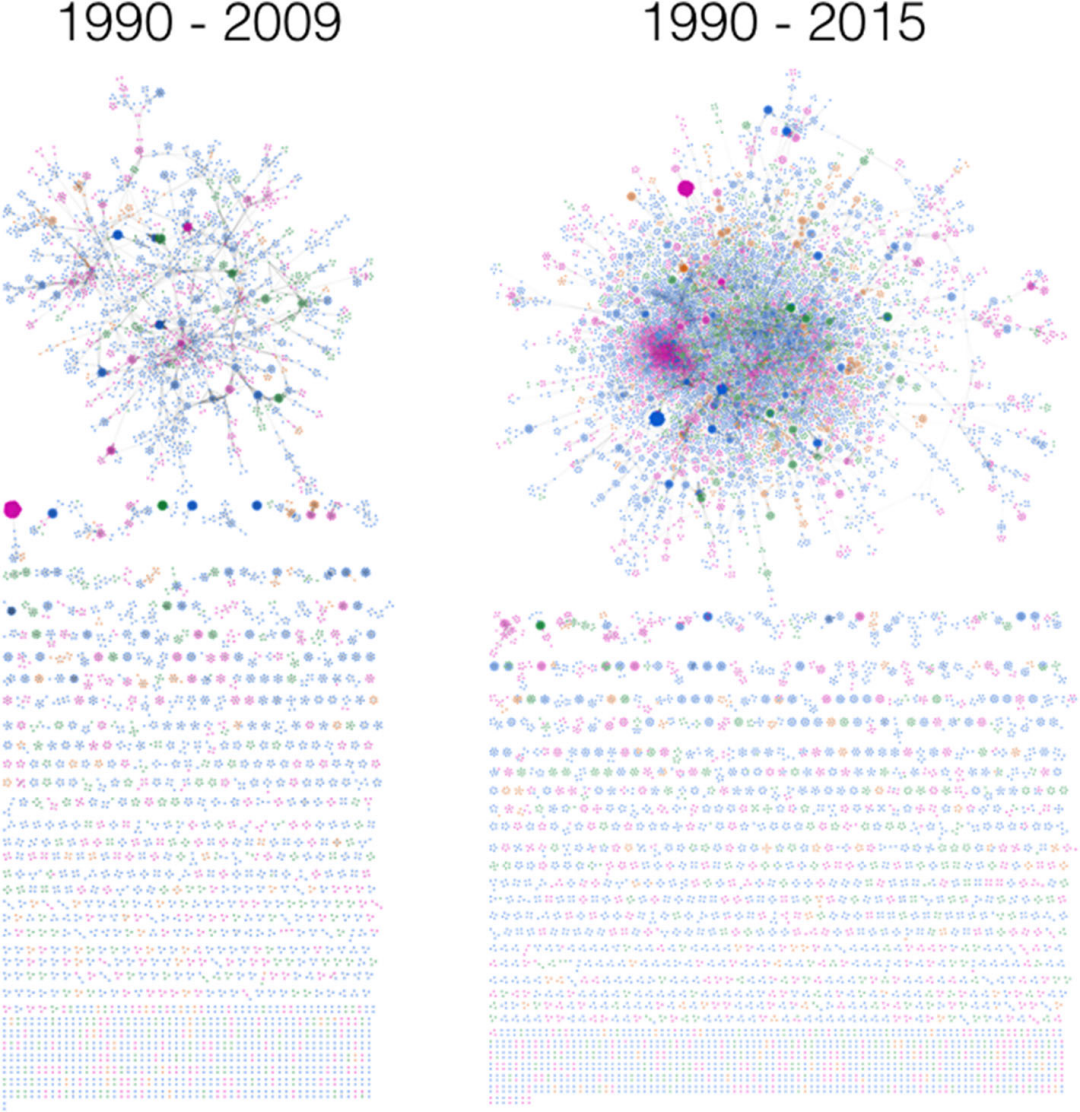
The structure of health policy and systems research co-authorship network from 1990 to 2009 (left panel) and 1990 to 2015 (right panel). Node colour represents first-authors’ economic region. Compared with the previous figures, the size, connectivity and robustness of the large connected component (LCC) grows over time. From 1990 to 2009 (left panel) 4375 nodes (65%) contribute to the formation of SDCs and 2394 nodes (35%) belong to the LCC, while during 1990–2015 (right panel) these are 6078 (39%) and 9623 (61%), respectively. *Orange colour* indicates low-income countries (LICs), *green* for lower middle-income countries (LowerMICs), *pink* for upper middle-income countries (UpperMICs) and *blue* for high-income countries (HICs)

The next cumulative interval between 1990 and 2009 captures the evolution of a more robust LCC, which is a result of expanding collaboration among a larger group of authors (2394 of 6769 nodes). The robustness of the network (Fig. 3, left panel) reached a level whereby the overall connectivity was not dependent on a few edges. However, despite the formation of a stable LCC, the majority of nodes (~65% or 4375 nodes) remain within SDCs.

**Fig. 4:**
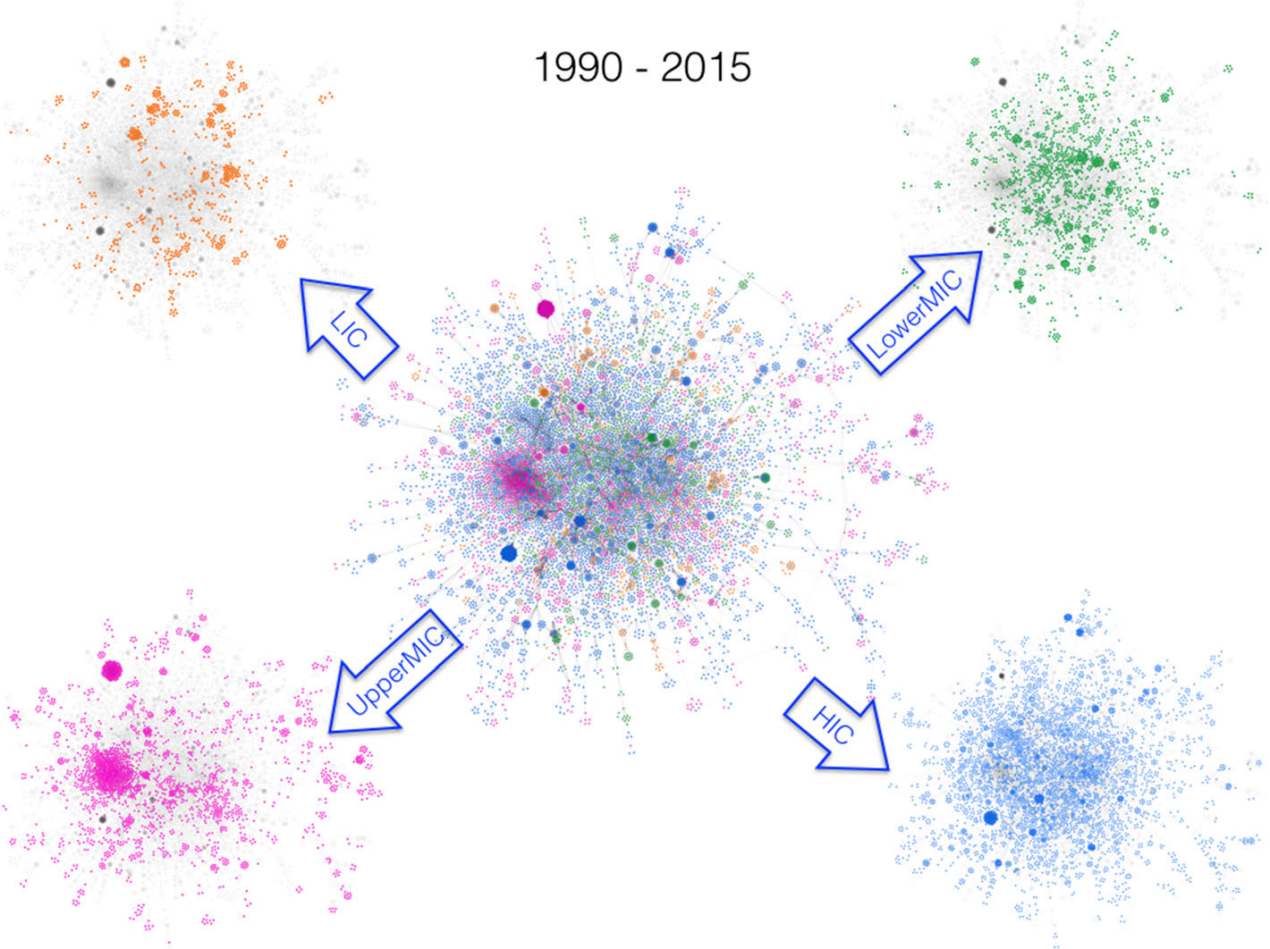
The large connected component (LCC) in the middle corresponds to the interval 1990–2015. The four surrounding networks (*grey* background) are identical to the one in the middle, but stratified by the economic classification of the first authors’ country affiliation. For description on the area marked by a dashed black circle, please see the next figure. *Orange colour* indicates low-income countries (LICs), *green* for lower middle-income countries (LowerMICs), *pink* for upper middle-income countries (UpperMICs) and *blue* for high-income countries (HICs)

During the last cumulative interval between 1990 and 2015 (Fig. 3, right panel), for the first time the number of nodes within the LCC (9623 or 61%) exceeds that corresponding to SDCs (6078 or 39%). The robustness of the network is indicative of the existence of multiple pathways between different groups and individuals, leading to cross-fertilisation of ideas and contribution of a broader group of experts from different disciplines to the HPSR literature. Stratification by region (Fig. 4) shows improvement in all economic regions.

**Fig. 5:**
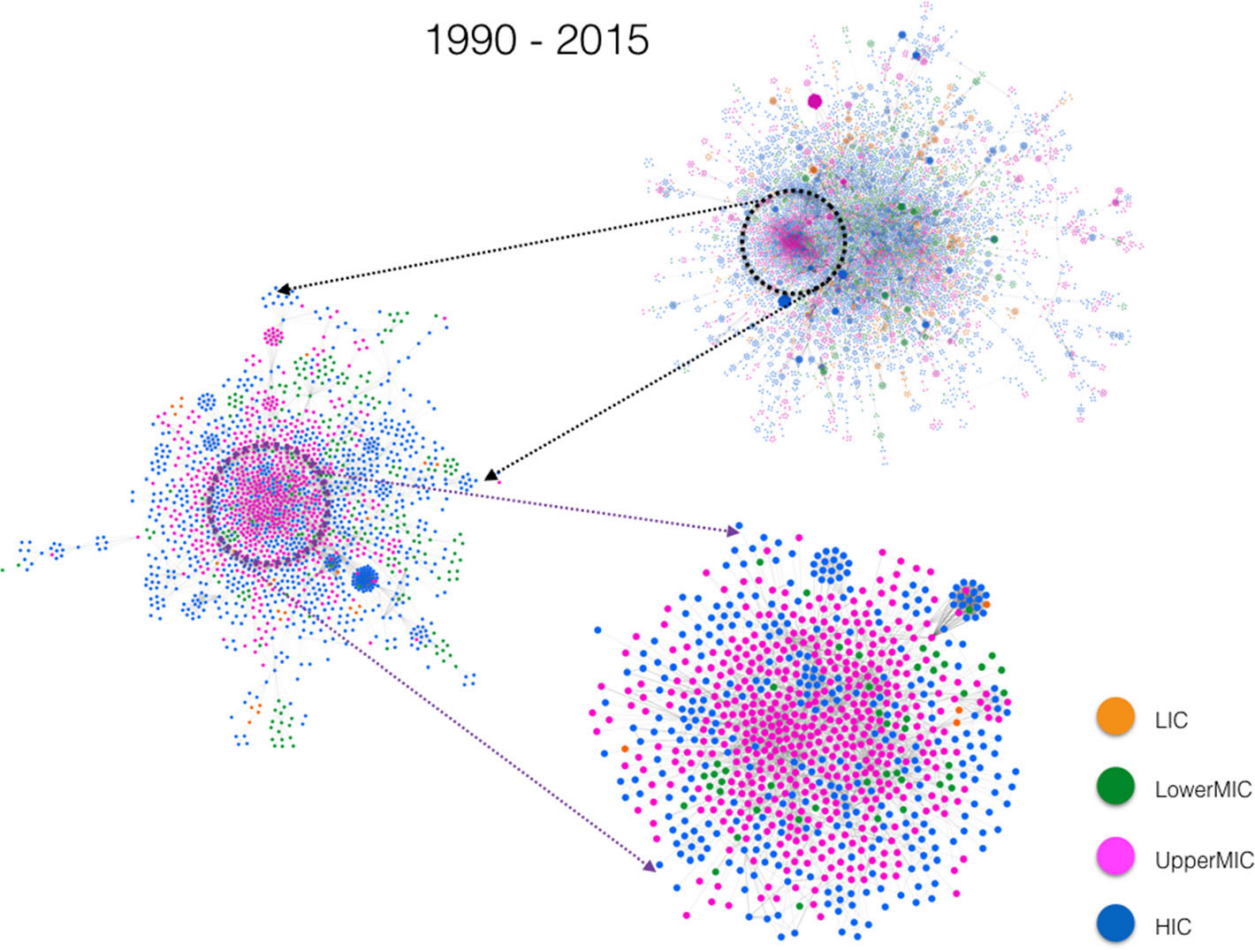
Successive magnification of a segment of the HPSR co-authorship network from 1990 to 2015 (starting from top right panel, to left, to bottom right panel) reveals a more intricate collaborative relationship between authors from different economic classifications. While in the previous cumulative intervals, authors from high-income countries used to play a dominant role in the overall connectivity of the network, the 1990–2015 cumulative interval shows that upper-middle-income countries (UpperMICs) are catching up in establishing their influence on the network. *Orange colour* indicates low-income countries (LICs), *green* for lower middle-income countries (LowerMICs), *pink* for UpperMICs and *blue* for high-income countries (HICs)

An important global feature of the 1990 to 2015 network is the emergence of a strongly connected cluster influenced by the UpperMICs (Fig. 5). This emergent pattern, predominantly driven by Brazil, China, South Africa, Iran and Thailand, has helped the UpperMICs to shape the global structure of the HPSR co-authorship network on par with HICs. More importantly, this emergent cluster also acts as a hub to connect authors from all economic regions (see the lower panel in Fig. 5). Among LowerMICs, the global spread is predominantly driven by India, Pakistan, Kenya and Nigeria.

Facilitating the growth of similar hubs in the years to come may considerably strengthen the global structure and robustness of the network, especially if it integrates, more profoundly, authorship from LICs and LowerMICs.

### HPSR literature by the numbers: co-authors, publications, citations

The co-authorship network may also be examined in terms of the authors’ collaborative reach, by considering their ‘degree’. A node’s degree is the number of edges emanating from it. In the context of a co-authorship network, a node’s degree is the overall number of other individuals with whom they co-authored. An author might have one or few publications co-authored with many people, thus a high degree. Alternatively, an author may have many publications co-authored with few individuals overall, then the node has a lower degree. It is also possible that an author has several publications co-authored with several people overall (high degree), or one has few publications with few people (low degree).

**Fig. 6:**
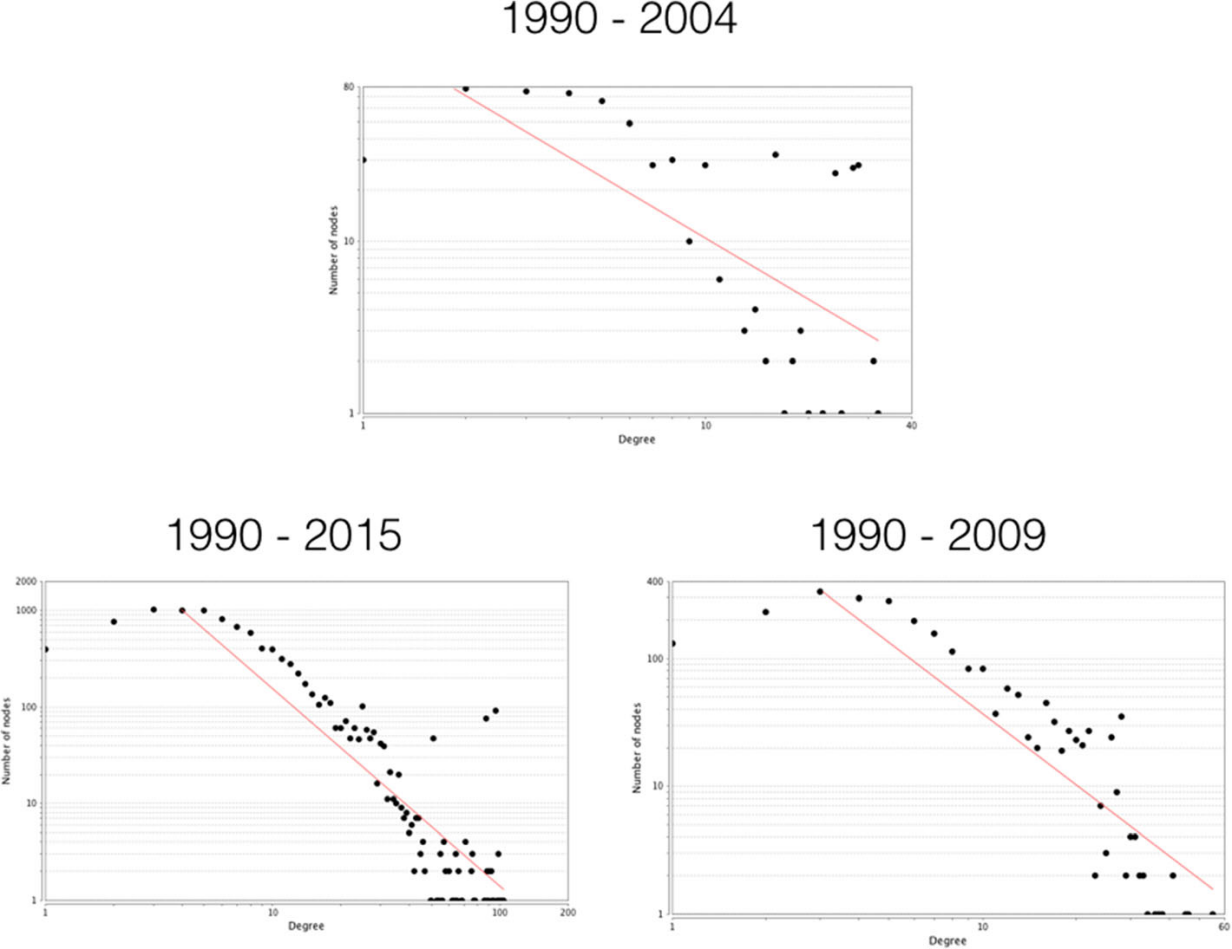
Degree distributions (*black dots*) of the large connected components corresponding to three cumulative time intervals. The best logarithmic fit to the data set in each panel is depicted by the *red line*

The frequency distribution of degrees for all nodes across the network is called the ‘degree distribution’ of that network. It is important to highlight that the degree only corresponds to the papers that satisfy our search criteria; thus, an author might have produced more papers in any given interval than shown, but these would be outside the HPSR scope of this analysis. Figure 6 shows the degree distributions of number of publications for the LCC of three networks introduced earlier (black dots). In these figures, both horizontal and vertical axes are in logarithmic scale, which allows values with different orders of magnitude to appear in one figure. Also shown in each panel is a fitted (red) line to data points. Such line on a logarithmic (log–log) plot is indicative of scale-free (or power law) distribution. In networks with scale-free distribution, a small fraction of nodes has very many contacts (right hand side of data points in each panel), while the majority of nodes have very few contacts (left hand side of data points in each panel).

Progressive examination of the three panels reveals that, generally, the same group of authors contribute to the right-hand of the distribution tails shown in Fig. 6. This conforms with the notion that ‘the rich get richer’, which is a generic feature of scale-free networks, and have been observed in a wide array of network structures representing natural and socio-technological systems. In the context of co-authorship networks, this implies that few groups/authors could establish themselves as key players by increasingly attracting relevant funds and human resources over time, to sustain their HPSR publication. While the establishment of strong hubs is generally viewed positively, at the global level, there is a risk of inadequate distribution of resources in regions where they are needed most. As such, it would be important to iteratively examine the future potential for new hubs to emerge in different socioeconomic regions.

In a co-authorship network, nodes may also represent the number of HPSR publications per author. In addition to the number of publications, it is also important to examine to what extent an individual’s work has had an impact on the scientific community. A measure used to evaluate this impact or influence is the number of times an author’s paper is cited. Since a network structure encapsulates information about all papers published by a person, a more appropriate measure is the total number of times that an author's papers are collectively cited up to the end date in each interval.

**Fig. 7:**
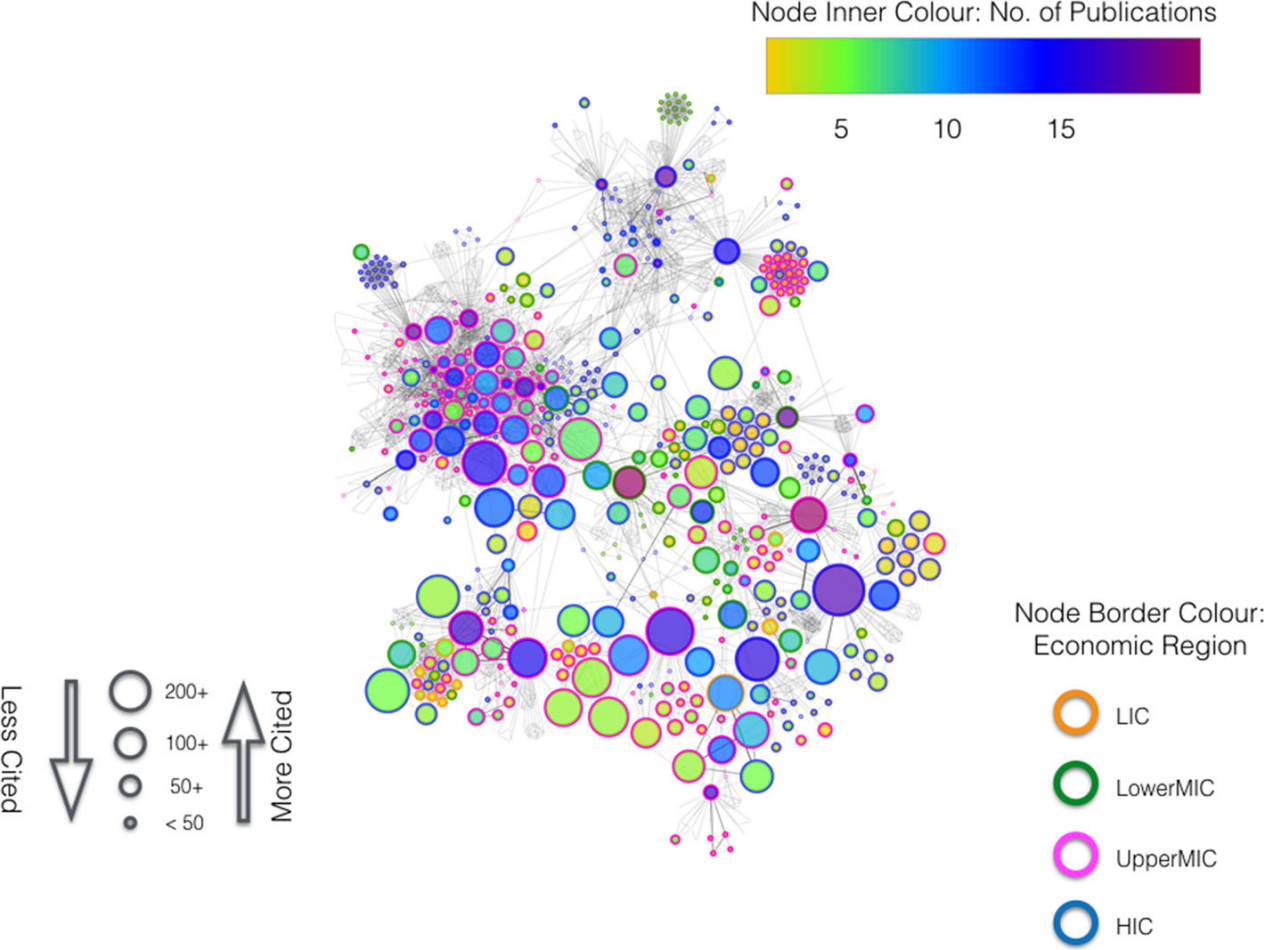
A subset of the 1990–2015 co-authorship network (n = 1026 nodes) that includes the most prolific authors (with 15 papers and more), as well as their first neighbours. The first neighbour of a node are those nodes that are directly connected to that original node by an edge. This figure is information rich and shows more attributes per node, including the number of publications (body colour) the number of times an author’s work is cited (size) and the first-author’s economic region (border colour). *Orange colour* indicates low-income countries (LICs), *green* for lower middle-income countries (LowerMICs), *pink* for upper middle-income countries (UpperMICs) and *blue* for high-income countries (HICs)

To examine number of publications and times cited more closely, we extract the most prolific HPSR authors (to the end of 2015) who published 15 HPSR papers or more, along with their first neighbours. The first neighbours of a node are the other nodes directly connected to the original node by an edge, regardless of their number of publications. This subset of 21 most prolific authors and their first neighbours leads to a network of 1026 nodes, which is shown in Fig. 7. In this figure, the node’s inner colour corresponds to the author’s number of publications (see figure legend), size corresponds to the number of times cited and border colour represents the first author’s economic region. One important feature observed from this network is that the number of publications does not necessarily correlate with number of times cited for an author. Another feature is that, by and large, highly-cited authors are from HICs or UpperMICs. Only a handful of top publishers and/or highly-cited individuals come from LowerMICs. Representation of LICs in this subset remains marginal.

In general, bibliometric analysis examines the frequency of publications over time. Co-authorship and citation analysis are an extension of this and are best understood using network analysis.

In this study, we used PubMed as the main database due to its comprehensiveness. This came at a limitation that only the affiliation of the first author of a paper was required for this dataset prior to 2014. Starting in 2014, PubMed has added subsequent authors’ affiliations to the database.

Availability of more refined data and resources in the future to include more country- and institution-specific information will allow us to capture more delicate patterns from the co-authorship. We did not include a list of most frequently published authors so as to avoid singling out individuals.

## Conclusion

Complexity science and network analysis add tremendous value to our understanding of the growth in HPSR. This analysis shows patterns of knowledge production (publication), collaboration (co-authorship) and potential policy influence (citation volume) over time and between countries. We consider that the bulk of citations may not necessarily be restricted to purely academic studies, as many indexed publications indeed stem from proceedings, reports, policy meetings, working groups, etc. This approach can identify and encourage support for regions with fewer publications and/or citations to increase participation and influence, as well as facilitating opportunities for collaboration across economic classifications to ensure LMICs meaningfully participate in HPSR.

This analysis summarised the evolution of the publication, co-authorship and citation networks within HPSR. Initially (early 1990s), groups operated almost exclusively independently and despite the topic being specifically on health policy in LMICs, HICs provided lead authorship. Since the early 1990s, the network has slowly but significantly evolved given the relatively short time period. In the full set analysis (1990–2015), for the first time in HPSR history, more than half of the authors are connected and lead authorship from UpperMICs is on par with that of HICs. This demonstrates the shift in participation and influence toward regions which HPSR primarily serves.

Enhancing support for participation by the LMIC that the discipline is meant to serve is imperative for success, and in particular LICs, since publications in these countries are increasing at a greater pace than any other economic region, but the absolute number is quite low. Thus, while capacity is expanding, additional support will greatly enhance this growth until they are more adequately represented within the discipline.

This study provides an unprecedented perspective and sheds light on the regional heterogeneity in contribution to HPSR, necessitating elevated investment for HPSR capacity-building in LICs and LowerMICs, facilitating UpperMICs to become more prominent players, and investing in moving away from a core-reliant co-authorship network structure towards a more distributed network structure.

## Abbreviations

GNI: gross national income
HPSR: health policy and systems research
LCC: largest connected component
LICs: low-income countries
LMICs: low- and middle-income countries
LowerMICs: lower-middle income countries
SDC: small disjoint chains
UpperMICs: upper middle-income countries
